# Effects of analytical and experiential self-focus on stress-induced cognitive reactivity in eating disorder psychopathology

**DOI:** 10.1016/j.brat.2011.06.011

**Published:** 2011-10

**Authors:** Adhip Rawal, J. Mark G. Williams, Rebecca J. Park

**Affiliations:** Department of Psychiatry, University of Oxford, OX3 7JX, UK

**Keywords:** Eating disorders, Anorexia nervosa, Rumination, Self-focus, Processing mode, Cognitive reactivity, Emotional processing

## Abstract

Previous research suggests distinct modes of self-focus, each with distinct functional properties: Analytical self-focus appears maladaptive, with experiential self-focus having more adaptive effects on indices of cognitive-affective functioning (e.g., Watkins, Moberly, & Moulds, 2008). The authors applied this framework to eating disorder (ED) psychopathology and manipulated the mode of self-focus prior to exposure to a stressor (imagining eating a large meal; Shafran, Teachman, Kerry, & Rachman, 1999). Study 1 showed that students high in ED psychopathology reported lower post-stressor feelings of weight or shape change and less subsequent attempts to neutralise (e.g., imagining exercising) after experiential relative to analytical self-focus. Study 2 found that partially weight restored patients with anorexia nervosa had lower post-stressor estimates of their own weight and reported lower urge to cancel stressor effects following experiential compared to analytical self-focus. Experiential self-focus was also followed by less neutralisation than analytical self-focus. Results suggest that the mode of self-focus affects cognitive reactivity following a stressor in individuals with ED psychopathology. Examining the mode within which individuals with ED psychopathology focus on self and body may raise important implications for understanding of psychopathology and open new possibilities for augmenting current treatments.

## Highlights

► The *way* individuals with eating disorders focus on self/body may maintain pathology. ► Two experiments to examine self-focus effects on reactions to an eating stressor. ► Analytic and experiential self-focus had distinct influences on stress reactivity. ► Analytic focus increases weight/shape distortions and elicits compensatory thoughts. ► Shifting focus to *experiencing* vs. *thinking about* self/body may improve outcome.

## Introduction

Much of mental experience is concerned with focussing on physical and psychological aspects of self. As the literature on cognitive biases in psychological disorders exemplifies (e.g., Mathews & MacLeod, 2005), there are differences between individuals in the subject of this mental activity. An emerging body of research has also highlighted differences in the *way* self-focus is engaged in, besides differences in its content. This research has raised the critical idea that it is not sufficient to consider self-focus as a single, monolithic state but that it may be necessary to discriminate between different types of self-focus. In particular, investigation of ways one can direct attention to subjective experience has suggested a distinction between two modes of self-focus: analytical/ruminative vs. experiential (Teasdale, 1999; Watkins & Teasdale, 2001, 2004).

*Analytical self-focus* (sometimes also referred to as conceptual-evaluative or abstract self-focus) is characterised by ‘thinking about’ the self, one’s emotions and body. In this mode of processing a focus on discrepancies between current and desired states, and their evaluation, is a prominent feature. Attention is often directed to the self in relation to the past or future. In contrast, *experiential self-focus* (also referred to as concrete self-focus) is characterised by direct, non-evaluative attention to present-moment subjective states (thoughts, feelings, sensations). These are experienced in their “raw” state as they occur and are not elaborated upon. Whereas the former corresponds to a narrative-based, conceptual mode of processing self-material (e.g., “Why do I feel this way” or “What does this mean?”), the latter corresponds to a sensory-based, mindful mode of processing characterised by focus on mental or bodily experience itself in its entirety (e.g., “How does this feel, right now?”).

Analytical and experiential self-focus can be seen as different ways of processing self-referent information. It has been suggested that under conditions of negative and relatively undifferentiated self-representations, and where there is a discrepancy between current and desired mental or physical states, self-focus can become maladaptive (Barnard, 2004; Carver, Lawrence, & Scheier, 1999; Carver & Scheier, 1990; Greenberg & Pyszczynski, 1986; Watkins, 2008). Specifically, such circumstances are postulated to stimulate sustained ruminative/analytical processing activity on negative cognitive-affective content, reinforcing their elaboration, which in turn is thought to contribute to the establishment of self-perpetuating processing cycles (Teasdale & Barnard, 1993). This suggests that the particular quality of self-focused attention may be central to emotional processing, particularly at times of distress (Teasdale, 1999).

Emotional processing is described as a process whereby emotional disturbances are absorbed and decline to the extent that other experiences and behaviour can proceed without disruption (Rachman, 1980). It has been suggested that analytical and experiential self-focus are mutually exclusive ‘modes of mind’ that influence the level of emotional processing that takes place (Teasdale 1999; Williams, 2008). In the analytical mode, the continued conceptual-evaluative processing of emotional material is likely to maintain emotional disturbances. On the other hand, the experiential mode disengages individuals from such patterns of thinking that fuel dysfunctional self-evaluation. At the same time, in the absence of conceptual-evaluative processing, sustained experiential processing of emotional material enables emotional and behavioural change by fostering non-reactive engagement, exploration and awareness. Thus, whereas the analytical mode is expected to impede effective emotional processing, the experiential mode is expected to facilitate such processing (Teasdale, 1999).

These theoretical ideas have received increasing support from studies that manipulate the mode of processing experimentally. An analytical mode of self-focus has been induced by instructing participants to “think about the *causes, meanings, and consequences* of their thoughts and feelings”. An experiential mode of self-focus can be induced by instructing participants to “focus attention on the *experience* of their thoughts and feelings” thus encouraging direct moment-to-moment focus (e.g., Watkins & Teasdale, 2004). Several experimental studies, primarily with depressed samples, support the hypothesis that the particular mode of processing adopted during self-focus differentially affects its cognitive and emotional consequences. For example, manipulating the mode of self-focus influences autobiographical memory specificity, problem-solving ability, and emotional recovery from a laboratory-induced negative event, where relative to analytical self-focus, experiential self-focus has more adaptive effects (Sanders & Lam, 2010; Watkins, 2004; Watkins & Moulds, 2005; Watkins & Teasdale, 2004).

Park and Barnard (2006), Park, Dunn, and Barnard (in press) have recently applied these ideas to Eating Disorder (ED). Park et al.’s account focuses on the mental processing activity underlying cognitive-affective content and thus emphasises not only the thoughts, feelings and bodily experiences that occur in eating psychopathology, but also *how* people *relate to* these experiences. A key feature of this framework is the suggestion that analytical thinking is characteristic of ED, in particular Anorexia Nervosa (AN), and contributes to the maintenance of core ED psychopathology. For example, analytical processing in the form of persistent self-evaluation in terms of eating, weight or shape reinforces the centrality of self-control. A relative absence of bodily and emotional experience is a consequence of analytical processing, as conceptual representations of the body are at the forefront (that is, *thoughts about* rather than *experiences of* the body). This effect may be particularly potent given the ego-syntonic nature of EDs, whereby individuals are motivated to control emotions and body weight.

This account predicts that shifting individuals away from an analytical into an experiential mode of processing will interrupt ruminative thinking and provide an opportunity for the direct processing and integration of bodily and emotional cues that were previously avoided. In this way, the experiential mode may foster emotional change and modifications in self-representations such that they are less likely to perpetuate negative processing cycles. In sum, Park et al.’s (in press) account predicts that an analytical mode of processing contributes to the maintenance of maladaptive cognitions, feelings and behaviours in EDs, whereas encouraging an experiential mode may allow for attenuation of such concerns by facilitating more effective emotional processing.

There are several reasons to suggest that it may be useful to think about EDs from a mode of processing perspective. It is known that core ED psychopathology involves preoccupation with self, particularly with eating, weight and shape concerns and their control (Fairburn, Cooper, & Shafran, 2003; Fairburn, Shafran, & Cooper, 1998). Moreover, ED-related concepts are of central importance to these individuals’ sense of self (Cooper & Hunt, 1998; Cooper & Turner, 2000; Rawal, Park, & Williams, 2010). Self- and body-dissatisfaction are common in EDs (e.g., Shafran, Cooper, & Fairburn, 2002) and indicate the presence of self-ideal discrepancies. Maladaptive self-representations in EDs may thus set the stage for sustained analytical processing activity.

There is evidence to suggest that analytical self-focus is common in EDs. Analytical processing involves conceptual/evaluative *thinking*, which necessarily removes the person from sustained direct experience. Rumination – an abstract style of repetitive thinking about generic aspects of self-experience – and avoidance/suppression of direct experiential states are seen as key markers of an analytical mode of processing (Teasdale, 1999; Williams, 2008). Studies have shown that individuals with ED-concerns score higher on both measures of rumination (particularly brooding which is often associated with maladaptive cognitive-affective consequences) and experiential avoidance compared to healthy controls even after controlling for depression and anxiety levels (Rawal et al., 2010; see also Wildes, Ringham, & Marcus, 2010). Clinical interviews reveal that patients with EDs ruminate about life events and attempt to control internal experiences (Serpell, Treasure, Teasdale, & Sullivan, 1999; Troop, Holbrey, & Treasure, 1998). This is line with research that shows reduced awareness of emotional and bodily states in EDs (Gilboa-Schechtman, Avnon, Zubery, & Jeczmien, 2006; Kucharska-Pietura, Nikolaou, Masiak, & Treasure, 2004; Wagner, Ruf, Braus, & Schmidt, 2003). Avoidance shows some overlap with thought suppression, and associations between rumination, suppression and ED psychopathology have been reported (Aldao & Nolen-Hoeksema, 2010). Moreover, results from Rawal et al. (2010) also indicate that ED psychopathology is associated with positive beliefs about rumination. Such beliefs are positively correlated with vulnerability, frequency and intensity of rumination (Moulds, Yap, Kerr, Williams, & Kandris, 2010; Papageorgiou & Wells, 2009).

Both rumination and avoidance have been associated with increased cognitive and emotional dysfunction (Hayes, Wilson, Gifford, Follette, & Strosahl, 1996; Nolen-Hoeksema & Morrow, 1993; Spasojevic & Alloy, 2001) and such tendencies are also correlated with the onset and maintenance of abnormal eating behaviours (Lyubomirski, Casper, & Sousa, 2001; Nolen-Hoeksema, Stice, Wade, & Bohon, 2007). Finally, teaching self-awareness based on principles of experiential processing is associated with improvements in self- and body-acceptance in patients with EDs (Rawal, Enayati, Williams, & Park, 2009).

The aim of the current investigation was to test the prediction of Park et al. (in press) that analytical as compared to experiential processing plays a role in maintenance of symptoms in individuals with ED psychopathology. Specifically, we sought to test whether prior manipulation of the mode of processing differentially affected cognitive reactivity to an ED-specific stressor (imagining eating a large, fattening meal) independent of differential effects on mood. Reactivity to specific vulnerability-provoking situations is strongly associated with psychopathology and can be used as an index of the extent of emotional processing (Rachman, 1980; Segal et al., 2006; Teasdale, 1999). Previous research in non-clinical samples has shown that analytical and experiential self-focus are associated with the level of stress-related reactivity following exposure to a failure experience (Watkins, Moberly, & Moulds, 2008). We predicted that in individuals vulnerable to ED, analytical processing would be associated with increased stress-induced cognitive reactivity compared to experiential processing.

Our stressor involved a slightly modified version of a procedure developed by Shafran, Teachman, Kerry, and Rachman (1999) where participants were asked to imagine eating a large, fattening meal (see Method for further details). Shafran et al. showed that this procedure produced cognitive and emotional reactions associated with EDs (e.g., feelings of weight gain, fatness, anxiety and guilt) and elicited an urge to engage in a corrective response (e.g., checking or neutralisation). Reactivity was not mediated by depression or obsessionality, suggesting that this distortion may be specific to eating pathology.

Neutralisation refers to an attempt to reduce, remove or compensate for the discomforting effect of an intrusive thought, image or impulse (Rachman, Shafran, Mitchell, Trant, & Teachman, 1996). Neutralisation is important because it implies a potent effect of thoughts and it may consolidate the belief that compensatory thoughts or acts are necessary to deal with emotional distress. We focused on cognitive forms of neutralisation as opposed to other forms of corrective action (e.g., weight or shape checking), as previous research indicates that neutralisation occurs more frequently and is considered as more effective than checking (Shafran et al., 1999), and thus may be of clinical significance. We suggest that neutralisation is likely to be exacerbated when processing material in a conceptual-evaluative way (i.e., during analytical self-focus) relative to an experiential mode.

We report two studies. The first is an analogue study, comparing experimental mode manipulations in a student population with different levels of weight and shape concerns (chosen as a first step because such participants are less likely to suffer from complicating and co-morbid factors; Holderness, Brooks-Gunn, & Warren, 1994). The findings from this study prompted a second study of a small number of inpatients with EDs, repeating the mode manipulation.

## Study 1

### Hypothesis

In individuals with a high level of ED psychopathology, an ED-specific stressor will elicit less dysfunctional reactions (ED-related thoughts and neutralisation) after experiential compared to analytical self-focus.

### Method

#### Design

The design was a 2 (group: high vs. low ED psychopathology) × 2 (condition: analytical vs. experiential) mixed factorial design. Participants completed both analytical and experiential self-focus manipulations, each on separate occasions.

#### Participants

Forty female student volunteers from the University of Oxford were recruited for this study. The Mini International Neuropsychiatric Interview (MINI; Sheehan et al., 1998) for DSM-IV was used to determine that participants were free of either current or past axis-I-disorder including ED. It was administered by one member of the research team with training in conducting clinical interviews. Four participants had to be excluded on this basis as the interviews indicated history of disorder (2 cases of anxiety disorder, 1 case of major depression, 1 case of ED). Of the remaining 36 participants who completed the study, those individuals falling into the bottom and top quartile on the Eating Disorder Examination-Questionnaire (EDE-Q: Fairburn & Beglin, 1994) were included in the analysis (9 participants in each group). Participants were 18–26 years of age (*M* = 20.33, SD = 2.11); see Table 1 for characteristics. Participants had no neurological or current physical illnesses and were un-medicated at the time of testing. Participants received book vouchers for participation.

#### Materials

##### Questionnaires

The Beck Depression Inventory (BDI) and the Beck Anxiety Inventory (BAI) were used to assess levels of depressive and anxiety symptoms, respectively (Beck, Epstein, Brown, & Steer, 1988; Beck, Ward, Mendelson, Mock, & Erbaugh, 1961). These 21-item self-report scales measure the presence and severity of depression and anxiety symptoms over the previous week. Their validity and reliability have been well established (Beck et al., 1988). Cronbach’s alpha coefficients were.84 and.88, respectively.

The EDE-Q was used as a self-report measure of ED psychopathology. It assesses behavioural and attitudinal features of EDs over the preceding 4 weeks and comprises a global index of ED psychopathology and four subscales: restraint, eating concerns, weight concerns and shape concerns. The questionnaire has good reliability and validity (Luce & Crowther, 1999; Mond, Hay, Rodgers, Owen, & Beumont, 2004). Cronbach’s alpha for the subscales ranged from.76 to.92 in the current study.

##### Self-focus manipulations

The analytical and experiential self-focus manipulation tasks (Watkins & Teasdale, 2004) have been designed to influence the quality of participants’ thinking. Each manipulation consists of a list of 28 items or ideas (e.g., “the physical sensations in your body”, “the way you feel inside”, “the amount of certainty you feel”, “how sad or happy you are feeling”, “how weak or strong your body feels right now”) that participants are instructed to concentrate upon. Participants work through the items sequentially and at their own pace. Participants were asked to spend more time on items that made more sense/were more meaningful to them. Both tasks use exactly the same items (in the same order) focussing on self, body state, or emotions. The only difference between the self-focus tasks lies in how to focus attention on these items, either encouraging an analytical (“think about the causes, meanings and consequences”) or an experiential (“focus your attention on your experience of”) mode of processing. The task is timed for 8 min and participants were asked to return to the beginning of the list (once again focussing on more meaningful items) if they had finished working through the entire list before the time was up.

##### Self-focus manipulation check

To examine whether the self-focus manipulations influenced the way and extent participants were focussing on themselves, ratings on three Visual Analogue Scales (VAS) from 0 to 100 were obtained after each of the manipulations (*analytical check*: proportion of thoughts concerned with trying to understand, explain, or make sense of things; *experiential check*: degree of focus on sensory experience; *self-focus check*: proportion of time spent focussing on self).

##### Mood

Participants rated current mood on 2 VAS from 0 to 100 before and after the self-focus manipulations on following dimensions: happiness and despondency.

##### Body mass index (BMI)

BMI [weight (kg)/height^2^ (m^2^)] was calculated based on participants’ reports of their height and weight.

##### Stress test

A slightly modified version of Shafran et al.’s (1999) imaginary meal task was used as a stressor. Participants were asked to think of a food they considered extremely fattening and to complete the sentence “I am eating……” once they had done this. The purpose of this was to help participants conjure up a vivid image of eating the “forbidden” food. Participants were instructed to imagine eating the food as vividly as possible. The length was standardised to 2 min and operationalised as a guided imagery task. Participants were given prompts at regular intervals once they had signalled to have a vivid picture of the food in mind (e.g., “Imagine what the food looks like in front you”, “Imagine what it smells like when you hold it to your nose”, “Imagine placing the food into your mouth”, “Imagine the flavours and texture in your mouth”, “Imagine how eating this food makes you feel”). Subsequently, we obtained ratings of following ED-related variables:

■ Weight estimate: “How much do you think you weigh right now?”
■ Likelihood of weight gain/shape change: “How likely do you feel it is (0–100%) that you gained weight/changed shape solely from thinking about eating the forbidden food and writing the sentence?”
■ Urge to reduce/cancel effects: “How strong is your urge (0–100%) to reduce or cancel the effects of thinking about the food and writing the sentence?”

Answers to the first question were used to calculate a weight difference score (i.e., the difference between pre-stressor self-reported weight and post-stressor weight estimate; both measurements were obtained in kg). We considered these outcome variables to be clinically important, as it is known that EDs are associated with distorted self-cognitions and irrational beliefs about the importance of thoughts about eating, weight and shape (Fairburn et al., 2003). Moreover, these are often triggered by eating experiences. On this basis, it seemed reasonable to assume that an imaginary meal may elicit distorted self-cognitions such as discrepant perception of body weight and the feeling that one has gained weight or changed shape. Such responses may play a role in the maintenance ED psychopathology.

##### Neutralisation

Self-reported neutralisation was assessed post-stressor. For this, participants were asked whether they had engaged in a cognitive form of neutralisation, which was explained to participants as an attempt to reduce or remove negative feelings triggered by the stress test (e.g., through a thought or image). We assessed neutralisation that occurred spontaneously and without prior suggestion. Based on this neutralisation was coded as present (=1) or absent (=0) for analysis (see Procedure for more details).

##### Imagery task checks

To assess whether the imagery task was experienced differently across conditions, ratings on three VAS from 0 to 100 for focus on food as an image (picture in mind), vividness/intensity of image, and extent of focus on the image for the duration of the imagery task were obtained.

#### Procedure

All participants were tested individually and took part in both conditions on two separate occasions. Informed consent was obtained prior to the first visit. The sessions took place a week apart from each other and the order of completion of conditions was counterbalanced (half of participants analytical first, half experiential first). The questionnaire measures and the MINI were spread over the two sessions and administered before the self-focus manipulations. Weight and height assessments were obtained during the MINI and completion of the EDE-Q. The MINI and EDE-Q were administered on separate occasions due to two reasons: a) in order to provide a measurement of self-reported current weight on each day of testing, which was used as the pre-stressor weight score, and b) in an attempt to avoid that potential changes in mood due to ED psychopathology assessment were systematically stronger on a particular day of testing.

After administration of questionnaires, the remaining tasks and assessments were completed in the following order: time 1 mood ratings, self-focus manipulation (analytical or experiential), time 2 mood ratings, self-focus manipulation checks, stress test (imaginary meal – administered during both days of testing), imagery task checks, and assessment of post-stressor reactions and neutralisation.

To assess neutralisation, participants were left on their own, unprompted, for 1 min following the completion of the ED-specific variables (weight estimate etc.). Upon return, the researcher asked participants whether they had engaged in neutralisation during this time (neutralisation was explained as described above). If this was the case, participants were asked if the neutralisation was related to the imaginary meal task and was designed to make them feel better (and did not occur due to other reasons, e.g., boredom). Exact verbatim details were as follows: *During the time that you were alone, were you thinking of or imagining something in order to help you reduce, remove or cancel out any negative feelings or distress from the imaginary meal task? If so, was this specifically to make you feel better? If you had a thought or image because of other reasons (*e.g., *because you were bored) this does not count*. Subsequently, participants were debriefed and thanked for participation.

#### Data analysis

Data were analysed by a series of *t*-tests and analyses of co-variance (ANCOVA). An alpha level of.05 was used for all statistical tests.

### Results

#### Comparison of participants in high and low ED groups

The groups were comparable in terms of age and BMI (*t*s < 1.24, *p*s > .23). As necessitated by the split of the sample, the high and low ED pathology groups differed significantly in terms of EDE-Q global and subscale scores (*t*s > 6.17, *p*s < .01, *d*s > 2.91; see Table 1; subscale scores are not reported in Table 1 but are available on request). The groups also differed in BDI and BAI levels (*t*s > 2.83, *p*s < .02, *d*s > 1.33). These were controlled for in following analyses.

#### Manipulation checks

##### Self-focus manipulation checks

As expected, significantly greater analytical thinking was reported after analytical than experiential self-focus, *F*(1,14) = 13.02, *p* < .01, *η_p_*^2^ = .48 (*M* = 72.67, SD = 11.88 vs. *M* = 58.06, SD = 23.08). Similarly, there was significantly greater focus on momentary sensory experience after experiential than analytical self-focus, *F*(1,14) = 11.78, *p* < .01, *η_p_*^2^ = .46 (*M* = 71.39, SD = 13.26 vs. *M* = 44.44, SD = 20.64). As predicted, there was no difference between the conditions in the degree of self-focus (*F* < 1). None of the group or condition × group effects were significant (*F*s < 1). In sum, although the experimental manipulations had equivalent effects on the amount of self-focus in general, they led to a differential increase in analytical vs. experiential self-focus ratings, as predicted.

##### Mood

There was a main effect of group on ratings of despondency, *F*(1,14) = 12.37, *p* > .01, *η_p_*^2^ = .44, which indicated greater levels of despondency in the high compared to the low ED concern group (*M* = 37.89, SE = 4.63 vs. *M* = 14.86, SE = 4.63). No other main effects or interactions were significant (*F*s < 2.58, *p*s > .13), suggesting no self-focus related effect on mood.

##### Imagery task checks

None of the effects for any of the variables were significant (*F*s < 2.41, *p*s > .13) showing that there were no systematic differences in the experience of the imagery task between groups or conditions.

#### Post-stressor ED-specific reactions

##### Self-report

Reactions to the stressor were analysed by separate 2 (group: high vs. low) × (condition: analytical vs. experiential) repeated measures ANCOVAs. Weight difference scores, ratings of likelihood of weight gain/shape change, and urge to reduce/cancel effects served as the dependent measures.

The analysis revealed a main effect of condition on weight difference scores (calculated by subtracting pre-stressor weight in kg from post-stressor weight estimate in kg; *F*(1,14) = 4.87, *p* = .05, *η_p_*^2^ = .26). Participants gave a reduced estimate of weight after experiential compared to analytical self-focus (*M* = −.11, SD = 1.02 vs. *M* = .28, SD = .74; see Table 2). The main effect for group and the group × condition interaction were not significant, *F*s < .72, *p*s > .40.^1^

We did not find group or condition main effects for ratings of likelihood of weight gain/shape change, *F*s < 3.22, *p*s > .09. However, there was a significant group x condition, *F*(1,14) = 7.72, *p* = .02, *η_p_*^2^ = .37. Paired comparisons showed that this was due to a greater likelihood of weight gain/shape change after analytical vs. experiential self-focus in the high, *t*(8) = 2.33, *p* = .05, *d* = .70 (*M* = 28.33, SD = 15.21 vs. *M* = 17.22, SD = 18.56; see Table 2) but not low ED group (*t*(8) = 1.00, *p* = .35).

The analysis also revealed a group main effect for urge to reduce/cancel effects of the imagery task, *F*(1,14) = 7.23, *p* = .02, *η_p_*^2^ = .34, with greater scores in the high ED compared to the low ED concern group (*M* = 40.39, SE = 5.61 vs. *M* = 10.56, SE = 5.61). However, neither the condition main effect nor the group × condition were significant, *F*s < .65, *p*s > .42.

##### Neutralisation

All instances of neutralisation were related to the stress test and were aimed at improving mood state. Reported neutralisation included thoughts such as eating lettuce or imagining exercising. The analysis showed an association between condition and the frequency of neutralisation (*p* < .01, *Fisher’s Exact Test*). Analysing the sample separated by group showed no association between the variables in the low ED group (2 out of 9 neutralisers after analytical self-focus vs. 0 out of 9 after experiential self-focus; *p* = .47, *Fisher’s Exact Test*). However, the variables were significantly associated in the high ED group (*p* < .01, *Fisher’s Exact Test*), indicating neutralisation was less likely to follow experiential than analytical self-focus (1 out of 9 neutralisers in the experiential vs. 8 out of 9 neutralisers in the analytical condition; see Table 2).

All analyses were repeated with the order of condition (analytical first vs. experiential first) as an additional variable. There were no main effects for order or interactions with experimental condition (*p*s > .17).

### Discussion Study 1

The aim of this experiment was to investigate the effects of analytical and experiential self-focus on stress-induced cognitive reactivity in groups of high and low ED psychopathology. The ED groups did not differ in age or BMI levels. As predicted, there were no differences between the self-focus conditions in terms of their effect on mood, the amount of self-focus, or in the overall quality of the imaginary meal tasks.

Results showed that analytic self-focus was followed by higher weight estimates compared to experiential self-focus in both groups. Experiential self-focus was also associated with lower likelihood of weight gain/shape change and less reported neutralisation than analytical self-focus in the high ED group only. However, no self-focus effects on the urge to reduce or cancel effects of the stressor were found.

While the findings from this study are not entirely conclusive, they are largely consistent with our hypothesis that analytical and experiential self-focus have distinct influences on stress-related cognitive reactivity in individuals with ED concerns, with analytical self-focus exacerbating ED symptoms.^2^

## Study 2

The second experiment aimed to provide some further evidence from a clinical sample. We recruited a small series of partially weight restored inpatients with AN for a within-subject design that was a procedural replication of Study 1.

### Hypothesis

We predicted less stress-induced reactivity (ED-related thoughts and neutralisation) after experiential compared to analytical self-focus in the ED group.

### Method

#### Participants

ED patients were recruited from the ED service for Oxfordshire & Buckinghamshire. This is a tertiary service for patients with severe EDs. Letters of invitation and information sheets were distributed to patients who were asked to return a reply slip to hospital staff if they were interested in taking part in the study. Twenty-one patients participated, although six patients did not complete both visits. A further two patients had to be excluded because their EDE-Q scores were outliers and skewed the distribution (defined as at least two standard deviations below the mean). Thirteen ED patients completed both visits (analytical, experiential).

All patients had a clinical diagnosis of AN at time of in-patient admission, as verified by independent clinical examination, although some had gained weight since then.^3^ Co-morbidity was frequent: Eight patients had a diagnosis of major depression, three of obsessive-compulsive disorder, three of generalised anxiety disorder, five of social phobia, and three of post-traumatic stress disorder. One patient also had a past diagnosis of bulimia nervosa. The MINI and Eating Disorder Examination (EDE; Fairburn & Cooper, 1993) were carried out to confirm diagnoses. Thirteen student volunteers from the University of Oxford were recruited as controls. The MINI was carried out to exclude current or history of psychiatric disorder. Control participants had no current physical illnesses and were un-medicated at the time of testing. Other exclusion criteria for this study were: active psychosis, any medical condition that significantly affected concentration, history of head injury or stroke, or any physical disability that impacted on body image. Overall, the sample consisted of 26 participants (13 ED patients, 13 healthy controls) matched for age, sex (all participants were female) and verbal-IQ (see Table 3). Age in the current sample ranged from 18 to 37 (*M* = 25.11, SD = 4.74). Participants received book vouchers for participation.

#### Materials

##### Clinical interview

The EDE (Fairburn & Cooper, 1993) is a semi-structured interview, which is considered the ‘gold standard’ interviewer-based measure of ED psychopathology. It was used to verify diagnosis of EDs in the patient group. The time frame of the EDE is the past 28 days, but diagnostic items encompass the previous three months. It has good internal consistency, convergent and divergent validity, test re-test and inter-rater reliability (e.g., Fairburn & Cooper, 1993; Rizvi, Peterson, Crow, & Agras, 2000).

##### Intelligence

The National Adult Reading Test (NART; Nelson, 1982) was used to assess participants’ verbal-IQ. The NART correlates significantly with scores of full scale, verbal and performance intelligence tests (Nelson & O’Connell, 1978), and was used to match the groups in terms of intellectual ability.

In addition to the above instruments, the following materials were used as in Study 1: the EDE-Q, BAI, BDI, MINI, VAS mood scales, self-focus manipulations and checks, as well as the same stress test and imagery checks. One modification to the stress test procedure was made. In Study 1, weight difference following the stressor was calculated by subtracting current weight from estimated post-stressor weight. It is possible that differences arose because weight estimations were not obtained pre-stressor (weight estimations are open to subjective influences and may differ from actual weight). Therefore, participants were asked to estimate current weight twice (pre- and post-stressor) and weight change was defined as the difference between these two measurements.

#### Procedure

The procedure was identical to the procedure for Study 1 with the following exceptions: The NART and EDE were included and administered before experimental manipulations. The EDE was administered by a member of the research team who had received training in its use. In all cases diagnosis was consistent with that established by hospital clinical staff. Participants participated in two sessions (analytical, experiential) on two separate occasions (which took place a week apart from each other). The order of conditions was counterbalanced.

#### Data analysis

Data were analysed by a series of *t*-tests and ANCOVAs. An alpha level of.05 was used for all statistical tests.

### Results

#### Comparison of ED patients and controls

The groups were comparable in terms of age and verbal-IQ (*t*s < .89, *p*s > .38; see Table 3). As expected, the ED patient group showed higher levels of ED psychopathology than controls in terms of the EDE-Q global and all subscale scores, all *t*s(1,24) > 7.81, *p*s < .01, *d*s > 3.18 (subscale means are not reported in Table 3 but are available on request). The ED group had a significantly lower BMI level than the control group, *t*(1,24) = 4.27, *p* < .01, *d* = 1.74, which showed that patients were underweight on average (patient range = 14.17–19.23; control range = 17.50–26.50).^4^ The patient group also displayed greater levels of depression, *t*(1,24) = 6.96, *p* < .01, *d* = 2.84, and anxiety, *t*(1,24) = 3.14, *p* < .01, *d* = 1.28, and thus both were entered as covariates in subsequent analyses.

#### Manipulation checks

##### Self-focus manipulation checks

There was a main effect of condition on ratings of analytical thinking, *F*(1,22) = 6.67, *p* = .02; *η_p_*^2^ = .23, which suggested significantly greater analytical thinking after analytical compared to experiential self-focus (*M* = 64.04, SD = 25.38 vs. *M* = 50.77, SD = 21.34).

Similarly, there was a main effect of condition on ratings of sensory focus, *F*(1,22) = 9.78, *p* = .01, *η_p_*^2^ = .31, which indicated greater focus on sensory experience after experiential self-focus compared analytical self-focus (*M* = 60.77, SD = 23.65 vs. *M* = 50.00, SD = 25.77). The analysis also showed a main effect for group, *F*(1,22) = 7.72, *p* = .02, *η_p_*^2^ = .26, which was attributable to higher levels of sensory focus in the control compared to the patient group (*M* = 73.11, SE = 7.31 vs. *M* = 37.66, SE = 7.31).

There was no difference in the degree of self-focus between the analytical and experiential conditions (*F* < .57). No further group effects or interactions were significant (all *F*s < 2.87, *p*s > .10). In sum, the analytical and experiential manipulations led to a differential increase in analytical thinking vs. sensory focus whilst having equivalent effects on the level of self-focus.

##### Mood

There was a main effect of group for happiness, *F*(1,22) = 4.50, *p* = .05, *η_p_*^2^ = .17, indicating higher levels of happiness (*M* = 5.00, SE = .45 vs. *M* = 3.33, SE = .54) in the control compared to the ED group. There were no other effects (*F*s < 2.54, *p*s > .12) suggesting no impact of self-focus manipulations on mood.

##### Imagery task checks

There was a main effect of group for vividness, *F*(1,22) = 5.32, *p* = .03, *η_p_*^2^ = .20, which indicated greater vividness in the control compared to the ED group (*M* = 8.35, SE = .56 vs. *M* = 6.08, SE = .56). No other main effects or interactions were evident (*F*s < 1.87, *p*s > .18), suggesting no differences in the experience of the imagery task across conditions.

#### ED-specific reactions to stress test

##### Self-report

Reactions to the stressor were analysed by separate 2 (group: ED patients vs. controls) × 2 (condition: analytical vs. experiential) repeated measures ANCOVAs. The same dependent measures as in Study 1 were used. Outcome measures were subjected to square-root transformation (no negative values were present) to normalise distribution.

**Fig. 1:**
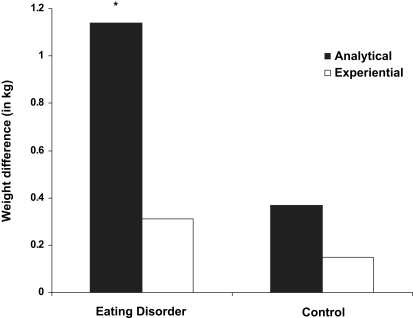
Change in weight estimate (in kg) following imaginary meal task separated by group and condition. * = Difference between analytical and experiential condition is significant (scores were subject to square-root transformation).

There was no main effect for group on weight estimate difference scores (*F* = 1.73, *p* = .20). We found a trend for a main effect of condition (*F* = 3.72, *p* = .07), which was qualified by a significant group × condition interaction, *F*(1,22) = 4.14, *p* = .05, *η_p_*^2^ = .16. Follow-up analysis showed that this was due to a significant difference in weight estimates (pre-weight estimate in kg subtracted from post-weight estimate in kg thus signalling increase in weight estimate following stressor) after analytical vs. experiential self-focus in the ED group, *t*(12) = 2.73, *p* = .02, *d* = .92 (*M* = 1.14, SD = 1.10 vs. *M* = .31, SD = .75), whereas there was no difference in the control group (*t*(12) = 1.59, *p* = .14, *M* = .37, SD = .60 vs. *M* = .15, SD = .38; see Fig. 1).

Analysis within conditions showed that ED patients’ weight estimate change in the analytical condition was also significantly different from zero (*t*(12) = 3.72, *p* < .01, *d* = .53), suggesting that patients were significantly overestimating their post-stressor weight. Equivalent comparison in the experiential condition was not significant (*t* = 1.48, *p* = .17). We also examined correlations between weight change scores and participants’ actual weight. This analysis showed a negative correlation between weight and weight estimate change scores in the analytic condition for ED patients (*r* = −.58, *p* = .04), suggesting that lower weight was associated with greater weight estimate changes. The correlation in the experiential condition was not significant (*r* = −.38, *p* = .20) and no associations were found in the control group (*r*s < .10, *p*s > .77). The pattern of results for the weight estimate variable was unaltered when using percentage change in estimated body weight as the outcome.

No main effects for group or condition on urge to reduce/cancel effects following the imaginary meal were found (*F*s < 1.05, *p*s > .31). However, the analysis revealed a significant group × condition interaction, *F*(1,22) = 5.31, *p* = .03, *η_p_*^2^ = .19. Follow-up analysis showed lower urge to reduce/cancel effects of the stressor after experiential self-focus compared analytical self-focus in the ED group, *t*(12) = 2.58, *p* = .02, *d* = .50 (*M* = 4.39, SD = 3.64 vs. *M* = 5.89, SD = 2.56). There was no difference between the conditions in the control group (*t*(12) = 1.59, *p* = .14; see Table 4). Correlations between participant weight and urge to reduce/cancel effect were not significant (*r*s < −.30, *p*s > .14).

There were no main effects or interactions for likelihood of weight gain/shape change, *F*s < 1.57, *p*s > .22. However, we did find a trend for a negative correlation between participant weight and ratings of weight gain/shape change in the analytic condition across both groups (*r* = −.37, *p* = .08), suggesting that lower weight was associated with higher scores on likelihood of weight gain/shape change.

##### Neutralisation

All instances of self-reported neutralisation were related to the stress test and were aimed at improving mood state. Data showed a significant association between condition and frequency of neutralisation (*p* = .05, *Fisher’s Exact Test*). Analysing the groups separately revealed a significant association between neutralisation and condition in the ED patient group (*p* = .04, *Fisher’s Exact Test*), but none in the control group (where no neutralisation occurred at all). In the ED group, neutralisation occurred less frequently following experiential self-focus compared to analytical self-focus (0 out of 13 neutralised after experiential self-focus, 5 out of 13 neutralised after analytical self-focus; see Table 4).

All analyses were repeated with order of condition as an additional variable. There were no main effects or interactions with experimental condition (*p*s > .18).

### Discussion Study 2

The aim of this experiment was to explore the hypothesis that analytical self-focus in patients with EDs would have more detrimental effects on emotional processing than an experiential mode of self-focus. Consistent with this hypothesis, results showed that ED patients had higher post-stressor estimates of their own weight following analytical self-focus and lower urges to cancel effects of the stressor following experiential self-focus. Moreover, reported neutralising cognitions were less frequent following experiential compared to analytical self-focus. However, no effects on likelihood of weight gain/shape change were found.

These findings await extension to larger samples. It should also be noted that EDE-Q levels in the control group were lower than existing norms for non-clinical groups (e.g., Mond et al., 2004) and comparisons to this group may have influenced results. Despite these limitations, results suggested that the mode of processing distinctly influenced the level of cognitive reactivity following an imaginary meal task in a sample of ED patients, consistent with predictions. Effects occurred in the absence of any impact of the self-focus manipulation on mood (replicating previous findings in depression; e.g., Watkins & Teasdale, 2004), or differences between the conditions in the experience of the imagery task, and when controlling for depression and anxiety levels. We did not covary BMI in this study based on the argument that this may contribute important variance to the effects of cognitive processing in ED patients (see 4). Correlations showed that weight was negatively associated with both weight estimate change and feelings of weight gain/shape change post-stressor in the analytic condition only. This suggests, as predicted by Park et al. (in press), that in individuals vulnerable to EDs lowered weight may amplify the effect of analytical processing on stress-induced cognitive reactivity. It should be noted that the overall pattern of results remained unchanged (although effect sizes were smaller) when we repeated analyses covarying for BMI, indicating that while weight status may exacerbate the effect of analytic processing, self-focus effects are not inherently attributable to BMI.

## General discussion

The aim of our experiments was to examine the effect of manipulating the mode of self-focus on stress-induced cognitive reactivity in individuals with ED psychopathology. To date, empirical studies of mode of self-focus have been primarily applied to depression. The present experiments extend this work to EDs and are the first to suggest that the consequences of self-focused thinking in EDs are moderated by the mode of processing, whereby an experiential mode is more adaptive relative to an analytical mode.

While not all variables were equally affected by the mode manipulation, the effects that we found were consistent with our hypothesis. Our outcome measures specifically tapped into beliefs associated with EDs (e.g., distorted weight perception, neutralisation). The finding that the mode in which information is processed moderates such outcomes after being exposed to a stressor is of clinical significance. Distorted perception about weight is argued to be involved in the maintenance of psychopathology (e.g., Vitousek & Hollon, 1990). Equally, neutralisation is important because it may represent a dysfunctional way of regulating distress, one that is likely to reinforce the over-evaluation of eating, weight and shape that is typically seen in EDs. Analytical processing is likely to exaggerate the tendency to focus on and evaluate self in the context of ED-related concepts. Consequently, the stress that eating causes may be exacerbated in this mode compared to experiential processing where it is likely to be less potent and ‘self-defining’. Processing emotional material in an experiential mode may thus weaken the connection between thoughts about eating and weight gain or neutralisation.

We assessed cognitive reactivity after exposure to an imaginary meal task, an experience that may resemble real-life eating situations which can often trigger psychopathology. This is because stress tests are likely to bring dysfunctional self-representations “online”, setting the stage for problematic responses. Previous research has shown that the extent of reactivity in response to challenges (e.g., induction of sad mood) predicts the persistence of psychopathology (Segal et al., 2006) and may be taken as index of the extent of emotional processing (Rachman, 1980; Teasdale, 1999). Our results suggest that the way individuals respond to such vulnerability-provoking situations may depend on the way self-material is processed, where analytical self-focus increases dysfunctional cognitive reactivity and impedes effective emotional processing compared to experiential processing which is likely to support more effective emotional processing. This effect may be due to the interruption of maladaptive cognitive-affective processing cycles, which serves to ‘inoculate’ vulnerable individuals from becoming engrossed in ED-related thoughts and feelings, thus allowing for alternative and less dysfunctional responses.

From a theoretical perspective, these results are consistent with Park et al.’s (in press) prediction of the distinct effects of analytical and experiential self-focus on ED psychopathology. This account specifically suggests that both cognitive content and process interact to determine outcome. It should be noted that most cognitive theories in EDs would predict that focussing on self and body would have negative consequences (e.g., Fairburn et al., 2003; Garner & Bemis, 1982). However, these theories cannot explain how processing the *same* material in *different* modes is associated with distinct outcomes. For example, whereas clinicians and researchers have identified over-focus on the body, shape and weight as a core feature of ED psychopathology, the core issue may be over-focus on *ideas about* the body rather than the *experience* of the body.

It is known that ruminative responses to situations are likely to maintain negative mood and dysfunctional attitudes (Nolen-Hoeksema, 1991; Nolen-Hoeksema & Morrow, 1993; Spasojevic & Alloy, 2001). Consequently, it has been suggested that interrupting ruminative processing cycles through shifting mode of self-focus may be an important factor for facilitating recovery from affective disorders and preventing relapse (Broderick, 2005; Crane, Barnhofer, Visser, Nightingale, & Williams, 2007; Segal, Williams, & Teasdale, 2002). Given the significance of this distinction for depression, it may turn out to be of importance for EDs as well. While the mode of self-focus does not explain the development of disorder-specific concerns, it may – if further substantiated – be an important process in the maintenance of dysfunctional thoughts across a range of psychopathologies, including EDs. Fostering experiential forms of self-awareness is one component of mindfulness-based interventions and initial evidence supports its effectiveness in treating EDs and for improving cognitive and behavioural flexibility (Merwin et al., 2011; Wanden-Berghe, Sanz-Valero, & Wanden-Berghe, 2011).

Our studies were the first empirical investigation of processing mode in EDs. As such, a number of issues require further inquiry. For example, it remains to be seen whether changes in processing mode can lead to enduring and clinically meaningful changes. One crucial prediction of the mode of processing framework is that it is the *interaction* of maladaptive cognitive-affective content and processing mode that is critical. While our findings were largely restricted to groups with ED pathology, our data do not prove that only those with ED pathology would show this effect. Demonstrating that the ED-specific stressor does not differentially affect responses of another psychiatric control group would be needed to substantiate this claim. For example, although depressed individuals are highly ruminative and show negative self-representations, the current stress task would be expected to show little effect in this group, as they differ in disorder-specific content. Moreover, our data do not show that stressors increase analytical thinking in EDs. Measuring momentary levels of analytical and experiential self-focus before and after the stress test would provide answers about the cause and effect of analytical processing. For example, verifying that stressors increase analytical self-focus in EDs (un-manipulated) and that this effect can be modified by manipulations would further support the utility of the mode of self-focus framework and for targeting the processing mode as a potential intervention.

A focus on both content and processing mode may also allow for a better understanding of potential peculiarities and implications of analytical processing in disorders such as EDs and depression. As described earlier, analytical self-focus involves ‘thinking about’ current and desired states of self. Unlike depression where current-ideal discrepancies and the criteria for closing this ‘gap’ are rather abstractly defined, the criteria used to make such judgements in EDs can be very concrete and measurable (e.g., weight change, waist size, or calorie intake). Therefore, rumination on generic aspects of self may be more characteristic of depression, whereas in EDs it may involve processing activity on more concrete features. Whereas concrete rumination has generally been thought of as more adaptive than abstract rumination (e.g., Watkins, 2008) as it aids problem-solving and goal progress, the suggestion here is that what *seems* to be concrete (e.g., shape or weight) is thought *about* rather than *experienced.* The point here is that anything can be processed analytically rather than experientially, and it is the *mode* with which it is processed that is critical, rather than the concreteness or abstraction of the referent itself.

It would be interesting to delineate the effect of self-focus on neutralisation further. Neutralisation effects were most consistent across both studies. As this was assessed following completion of other outcome measures and a brief ‘pause’ period, it may suggest that the effects of emotionally evocative events and their moderation unfold over time. As such, it would be interesting to examine cognitive reactivity over longer time periods. It may also be of value to assess other forms of neutralisation (e.g., crossing out or re-phrasing sentences) and to include checking behaviours (weight or mirror checking) to unpick this effect further. Checking behaviours are associated with ED concerns (e.g., Cooper, Deepak, Grocutt, & Bailey, 2007; Shafran et al., 1999), and acting on the basis of stress-related reactivity may reinforce a maladaptive cognition-behaviour link. This would also be useful as EDs are related to deficits in interoceptive awareness (e.g., Kucharska-Pietura et al., 2004), implying that self-report assessments may be problematic. Psychobiological measures may also usefully extend our studies, as emotional reactivity is expressed in various output systems which may be differentially affected.

Finally, some specific limitations of our studies need consideration. First, our sample requires some consideration. Our clinical ED sample consisted of individuals with partially weight restored AN. We were not able to quantify the degree of weight restoration and it is not clear what influence treatment and weight gain have on the processing of self-material (assessment of BMI levels at entry point and duration of EDs may prove important). Park et al. (in press) suggest that ruminative processing and its effects are exacerbated by low weight in those with EDs, which fits with clinical observations of worsening pathology with lower weight. Although our results suggested some influence of weight status on the effects of self-focus in this direction, it remains to be systematically examined whether self-focus effects are related to reductions in weight levels.

Generally, in-patient populations represent a minority of AN cases, indicating a particularly severe ED, which may impact on generalisability of our findings. For example, it is known that EDs are associated with rigid thinking styles (see Roberts, Tchanturia, Stahl, Southgate, & Treasure, 2007), which may be further compounded in severe cases. Cognitive inflexibility may influence the degree to which individuals are able to shift into an experiential processing mode. Therefore, concurrent training in cognitive flexibility may aid experiential processing manipulations.

Second, the current self-focus manipulations were developed for dysphoric people with much less psychopathology than individuals with clinical EDs. Given the tendency towards avoidance in ED samples, stronger effects may be obtained with more extensive experiential training procedures, or in those with less psychopathology.

Third, it is unclear from our data whether the analytic condition worsened reactivity or the experiential condition improved it. To clarify this, a neutral filler condition would have to be used. However, because each participant was their own control, we can say that the manipulation made a difference to reactivity to the stressor, and that this reaction did not seem to be mediated by difference in imageability of the food, or differences in mood. At this early stage in studying the impact of mode of processing, these are encouraging results that merit further investigation. One would expect potentially beneficial effects of distraction tasks to be short-lived and that individuals would revert to habitual, maladaptive processing styles, whereas an experiential mode may provide a beneficial form of self-focus.

Finally, we used a within-subjects design and it is possible that participants were aware of the crucial difference between the manipulations. While no participant correctly guessed the hypothesis, it is still possible that demands effects affected the response pattern.

In sum, while much work remains outstanding, our findings suggest distinct influences of the mode of processing on cognitive reactivity induced by an imagined eating stressor. These findings have clinical relevance for ED. The mode of processing analysis might augment understanding of psychological treatment and theory of EDs, firstly, by enhancing understanding of how effective treatments work, and secondly by providing a rationale for focussing on the processes – as well as content – underlying maintenance of psychopathology. In particular, the results add weight to the suggestion that interventions targeting analytical, ruminative processing in ED may augment existing interventions (Park et al., in press). Cognitive treatment for EDs has shown benefit to a wide range of patients with EDs but has limitations, particularly in the treatment of severe AN (Berkman, Lohr, & Bulik, 2007; Wilson, Grilo, & Vitousek, 2007). Notably, content-focused treatment approaches alone may not be effective in some individuals with EDs (Vanderlinden, 2008). Positive beliefs about EDs and its symptoms are common (Serpell et al., 1999) and many afflicted individuals seem resistant to changing thought content directly, or find this extremely difficult. Specifying aspects of process more explicitly may allow key phenomenology to be modelled more fully in order to develop more effective adjunctive strategies for treating ED psychopathology.

## Figures and Tables

**Table 1:** Sample characteristics Study 1.

	Low ED *(N = 9)*		High ED *(N = 9)*	
Variable	*M*	SD	*M*	SD
Age	20.78	2.28	19.89	1.96
EDE-Q Global^a^	.44	.24	3.73	.61
BMI	20.06	1.38	20.97	1.73
BDI^a^	4.78	3.67	15.11	6.17
BAI^a^	3.22	2.64	11.56	8.40

*Note*: EDE-Q = Eating Disorder Examination-Questionnaire; BMI = Body Mass Index; BDI = Beck Depression Inventory; BAI = Beck Anxiety Inventory.

a Group difference is significant.

**Table 2:** Means and standard deviations for self-focus manipulation checks, mood, imagery checks, and post-stressor ED-specific reactions separated by group and condition.

	Low ED group (*N* = 9)				High ED group (*N* = 9)			
	Analytical		Experiential		Analytical		Experiential	
Measure	*M*	SD	*M*	SD	*M*	SD	*M*	SD
Manipulation check								
Analytical thinking^a^	68.89	10.38	50.00	21.79	76.44	12.64	66.11	22.61
Sensory focus^a^	45.56	24.04	73.33	12.99	43.33	18.03	69.44	14.02
Self-focus	85.11	9.81	85.00	14.36	85.44	10.16	86.11	12.69
Mood								
Happiness, Time 1	68.56	12.07	66.22	12.47	52.11	27.84	60.00	11.97
Happiness, Time 2	58.11	20.71	64.00	11.59	48.67	28.23	56.22	13.66
Despondency Time 1	13.22	19.81	16.67	17.82	39.00	25.62	37.89	23.42
Despondency Time 2	11.89	18.94	17.67	16.51	35.56	21.85	39.11	18.21
Imagery check								
Image focus	75.89	10.61	75.56	14.93	71.56	10.65	71.22	7.31
Vividness	72.44	13.69	76.11	13.93	72.22	7.31	73.22	1.14
Duration	73.11	20.44	74.78	19.25	69.33	9.19	70.78	12.18
Stressor reaction								
Weight difference score^a^	.02	.07	.00	1.00	.53	1.00	−.22	1.09
Likelihood weight gain^c^	8.89	15.37	11.11	20.88	28.33	15.21	17.22	18.56
Urge reduce/cancel^b^	11.19	16.16	10.00	18.03	42.22	31.93	38.56	21.71
	*N*	%	*N*	%	*N*	%	*N*	%
Neutralisation^d^								
Yes	2	77.8	0	0	8	88.9	1	11.1
No	7	22.2	9	100	1	11.1	8	88.9

*Note*: Time 1 = pre-self-focus manipulation; Time 2 = post-self-focus manipulation; Weight difference score = post-weight estimate in kg minus self-reported pre-weight in kg.

a Condition main effect.

b Group main effect.

c Effect of condition in high ED group (group × condition interaction).

d Association between frequency of neutralisation and condition in high ED group.

**Table 3:** Sample characteristics Study 2.

	ED Patients *(N = 13)*		Controls *(N = 13)*	
Variable	*M*	SD	*M*	SD
Age	24.46	4.74	25.77	4.85
EDE-Q Global^a^	4.44	1.19	.53	.47
BMI^a^	17.16	1.61	21.06	2.87
BDI^a^	29.38	13.05	3.92	1.85
BAI^a^	21.15	13.99	7.00	8.29
Verbal-IQ (NART)	119.77	3.39	121.00	3.70

*Note*: EDE-Q = Eating Disorder Examination-Questionnaire; BMI = Body Mass Index; BDI = Beck Depression Inventory; BAI = Beck Anxiety Inventory; NART = National Adult Reading Test.

a Group difference is significant.

**Table 4:** Means and standard deviations for post-stressor ED-specific reactions separated by group and condition.

	ED patient group (*N* = 13)				Control group (*N* = 13)			
	Analytical		Experiential		Analytical		Experiential	
Measure	*M*	SD	*M*	SD	*M*	SD	*M*	SD
Stressor reaction								
Likelihood weight gain	4.75	2.98	2.68	2.83	.24	.88	1.18	1.98
Urge reduce/cancel^a^	5.89	2.56	4.39	3.64	.73	1.89	1.95	3.04
	*N*	%	*N*	%	*N*	%	*N*	%
Neutralisation^b^								
Yes	5	38.5	0	0	0	0	0	0
No	8	61.5	13	100	13	100	13	100

*Note*: Stressor reactions (self-report) on Visual Analogue Scales from 0 to 100. Stressor reaction ratings were subject to square-root transformation.

a Effect of condition in ED group (group × condition interaction).

b Association between frequency of neutralisation and condition in ED group.

## Footnotes

1 Single sample *t*-tests showed that the differences within conditions were not significantly different from zero (*p*s > .15). Moreover, correlations between pre-stressor weight and weight difference scores in each self-focus condition showed that weight change was not systematically related to participants’ actual weight (*r*s < −.27, *p*s > .29). Finally, the pattern of results was identical when analysing percentage change in estimate of body weight as the outcome.

2 Given the small sample size, we repeated the analysis using a median split on the EDE-Q to group participants into high and low ED psychopathology groups (18 participants in each group). While the findings were not as robust, the pattern of results was largely similar to the quartile split analysis: 1) There was still a main effect of condition for weight estimate difference scores (*F* = 4.08, *p* = .05, *η_p_* = .11), 2) there was a trend-level group × condition interaction for likelihood of weight gain/shape (*F* = 3.75, *p* = .06, *η_p_* = .10), 3) the effect on neutralisation was replicated, showing an association between condition and the frequency of neutralisation in the high ED pathology group only (*p* < .01), where 12 out of 18 participants showed neutralisation after the stressor in the analytic condition compared to 1 out of 18 in the experiential condition. These effects occurred in the absence of group differences in depression, anxiety, or BMI.

3 At time of testing, 5 patients’ BMI was above the formal cut-off for AN of 17.5 because they were on an intensive weight restoration programme. These were not excluded from the sample, as psychopathology was as severe as those of other patients who fulfilled all diagnostic criteria. However, technically speaking 5 out of the 13 patients had sub-threshold AN at time of testing. We therefore describe our clinical sample as patients with EDs or partially weight restored AN. One participant in the control group had a BMI of 17.5. This participant was not excluded as there was no other evidence of an ED on any psychopathology measure – interview or questionnaire. See Results for BMI range for both groups.

4 We did not covary BMI because this can be seen as (partly) an intrinsic feature of the difference between ED patients and controls. Both patterns of thought and behaviour are argued to be related to weight status (e.g., Fairburn et al., 1998; Park et al., in press). Thus, lowered weight may influence pathology and the effect of cognitive processing in EDs, suggesting that BMI contributes variance to the effects of self-focus on psychopathology.
